# Supplementary material for: The Application of Bony Labyrinth Methods for Forensic Affinity Estimation
Source: Biology (Basel). 2022 Jul 21;11(7):1088. doi: 10.3390/biology11071088 (PMC9312872; doi:10.3390/biology11071088)
Supplement: Supplementary file 1 [file biology-11-01088-s001.zip › biology-1811927-supplementary.pdf]

biology

MDPI

Supplementary Materials

The Application of Bony Labyrinth Methods for Forensic Affinity Estimation

Alexandra Uhl, Fotios Alexandros Karakostis and Katerina Harvati

Table S1. Measurement abbreviation list of the bony labyrinth semicircular canals and cochlea*. All measurements were taken in mm.

Abbreviation	Measurement
ASChM	Anterior Canal height
ASCwM	Anterior Canal width
ASCMR	Anterior Canal mean radius
ASChw	Anterior Canal height/width
LSChM	Lateral Canal height
LSCwM	Lateral Canal width
LSCMR	Lateral Canal mean radius
LSChw	Lateral Canal height/width
PSChM	Posterior Canal width
PSCwM	Posterior Canal width
PSCMR	Posterior Canal mean radius
PSChw	Posterior Canal height/width
ChM	Cochlear height
CwM	Cochlear width
CMR	Cochlear mean radius
ChwM	Cochlear height/width
SLI	Sagittal Labyrinthine index (SLIs+SLIi x100)

*For more details on measurements please see [18, 28].

Table S2. Error test results of raw measurements in mm. Five individuals were measured three times each.

Variable	Average Error	S.D.	Percent Error
ASCh	5.96	0.09	1.51
ASCw	6.72	0.05	0.76
ASCdh	6.69	0.10	1.47
ASCdw	6.40	0.07	1.06
PSCh	6.33	0.19	3.04
PSCw	5.80	0.05	0.85
LSCh	4.73	0.06	1.36
LSCw	4.94	0.08	1.55
Coh	5.10	0.17	3.35
Cow	4.32	0.08	1.89
SLIs	3.08	0.03	0.85
SLIi	2.88	0.04	1.28
